# Supplementary material for: Organic matter degradation causes enrichment of organic pollutants in hadal sediments
Source: Nat Commun. 2023 Apr 10;14:2012. doi: 10.1038/s41467-023-37718-z (PMC10086072; doi:10.1038/s41467-023-37718-z)
Supplement: Supplementary file 1 — Supplementary Information [file 41467_2023_37718_MOESM1_ESM.pdf]

Supplementary Information for

Organic matter degradation causes enrichment of organic pollutants in hadal sediments

Anna Sobek[1*], Sebastian Abel[1], Hamed Sanei[2], Stefano Bonaglia[3], Zhe Li[1], Gisela Horlitz[1], Arka Rudra[2], Kazumasa Oguri[4,5], Ronnie N Glud[4,6,7]

[1] Department of Environmental Science, Stockholm University, 10691 Stockholm, Sweden

[2] Lithospheric Organic Carbon (LOC) Group, Department of Geoscience, Aarhus University, Høegh-Guldbergs gade 2, 8000C, Aarhus, Denmark

[3] Department of Marine Sciences, University of Gothenburg, Gothenburg, Sweden

[4] HADAL and Nordcee, Department of Biology, University of Southern Denmark, Campusvej 55 5230 Odense, Denmark

[5] Research Institute for Global Change, Japan Agency for Marine-Earth Science and Technology, 2-15 Natsushima-cho, Yokosuka, 237-0061 Japan

[6] Danish Institute for Advanced Study (DIAS), University of Southern Denmark, Campusvej 55, 5230 Odense, Denmark

[7] Department of Ocean and Environmental Sciences, Tokyo University of Marine Science and Technology, 4-5-7 Konan, Minato-ku, Tokyo, 108-8477 Japan

* Corresponding author: anna.sobek@aces.su.se

Contents:

6 Pages

5 Tables

1 Figure

Supplementary Table 1. Total organic carbon content (TOC; %), protohydrocarbons (S1; mg HC g^{-1}) and Inert Fraction (%) in Atacama sediment samples. The presented data is the average of parameters determined in 10 mm sediment slices.

	**Sediment depth (mm)**	**TOC (%)**	**S1 (mg HC g^{-1})**	**Inert Fraction (%)**
**Atacama 1 (2560 m)**	0-20	2.18	0.68	72
	20-40	2.28	0.60	72
	40-60	2.22	0.67	71
	60-80	2.30	0.60	70
	80-100	2.06	0.55	70
**Atacama 9 (4050 m)**	0-20	0.97	0.19	80
	20-40	1.01	0.24	79
	40-60	1.02	0.16	80
	60-80	1.20	0.15	80
	80-100	1.34	0.23	80
**Atacama 6 (7720 m)**	0-20	0.67	0.07	86
	20-40	0.53	0.04	87
	40-60	0.52	0.04	87
	60-80	0.53	0.03	87
	80-100	0.50	0.05	86
**Atacama 2 (7995 m)**	0-20	0.60	0.08	89
	20-40	0.52	0.05	89
	40-60	0.62	0.10	87
	60-80	0.83	0.16	84
	80-100	0.87	0.21	83
**Atacama 4 (8085 m)**	0-20	0.60	0.07	87
	20-40	0.60	0.05	88
	40-60	0.58	0.05	89
	60-80	0.59	0.04	90
	80-100	0.61	0.06	86

Supplementary Table 2. Concentrations of polychlorinated biphenyl (PCB) congeners (pg g^{-1} dry sediment weight) in sediment from the Atacama trench area, where n.a. indicates no peak could be integrated and n.d. indicates the concentration was below detection limit. In addition, PCB congeners #170 and #209 were analyzed but not detected in any of the samples.

	**Sediment depth**	**#110**	**#118**	**#128**	**#136**	**#138**	**#149**	**#153**	**#180**	**#187**
	**mm**	**pg g^{-1} dwt**	**pg g^{-1} dwt**	**pg g^{-1} dwt**	**pg g^{-1} dwt**	**pg g^{-1} dwt**	**pg g^{-1} dwt**	**pg g^{-1} dwt**	**pg g^{-1} dwt**	**pg g^{-1} dwt**
**Atacama 1 (2560 m)**	0-20	1.77	1.95	1.29	n.d.	6.38	3.52	10.30	4.52	2.30
	20-40	1.54	1.54	0.81	0.56	4.21	2.51	7.35	2.62	1.40
	40-60	1.19	0.90	0.47	0.35	2.30	1.79	4.46	1.93	0.88
	60-80	1.07	0.87	0.33	0.43	1.72	1.41	4.71	1.05	0.50
	80-100	0.77	0.47	0.21	n.d.	0.94	0.91	3.07	0.56	0.26
**Atacama 9 (4050 m)**	0-20	1.03	1.55	n.a.	n.a.	3.11	2.13	13.70	1.78	1.51
	20-40	0.64	n.a.	n.a.	n.a.	1.63	0.95	4.35	1.63	0.77
	40-60	0.75	0.78	0.35	n.a.	2.30	1.32	2.77	1.14	0.62
	60-80	0.17	n.a.	0.18	n.a.	0.75	0.87	2.72	0.83	n.a.
	80-100	1.71	1.21	0.39	n.a.	n.a.	2.46	3.85	1.48	0.63
**Atacama 6 (7720 m)**	0-20	1.51	1.86	0.91	n.d.	5.09	2.99	9.99	4.27	1.84
	20-40	1.22	0.84	0.31	0.34	1.09	1.19	2.27	0.67	0.31
	40-60	0.66	n.d.	0.20	n.d.	0.99	0.92	22.40	0.97	0.32
	60-80	0.88	n.d.	0.28	n.d.	1.05	1.01	7.09	1.00	0.30
	80-100	n.d.	n.d.	0.22	n.d.	0.99	0.94	5.41	0.74	0.21
**Atacama 2 (7995 m)**	0-20	1.81	1.78	0.72	n.a.	1.92	2.64	4.81	3.50	1.31
	20-40	0.70	0.59	0.20	n.a.	1.26	0.93	2.34	0.79	0.39
	40-60	0.72	0.58	n.a.	0.25	0.62	1.04	2.09	0.83	0.25
	60-80	n.a.	n.a.	n.a.	n.a.	n.a.	n.a.	n.a.	n.a.	n.a.
	80-100	0.84	0.91	n.a.	n.a.	0.86	1.24	2.66	0.99	0.33
**Atacama 4 (8085 m)**	0-20	2.02	2.50	1.24	0.83	6.02	3.95	41.20	5.45	2.58
	20-40	1.20	n.d.	0.51	0.33	2.52	1.74	8.55	2.35	0.98
	40-60	n.d.	n.d.	n.d.	n.d.	n.d.	1.53	9.11	n.d.	0.36
	60-80	0.69	n.d.	0.21	0.31	1.02	0.87	9.51	0.99	0.23
	80-100	0.51	n.d.	0.23	n.d.	0.81	0.79	4.82	0.81	0.18

Supplementary Table 3. MSMS transitions for analyzed polychlorinated biphenyl (PCB) congeners and their internal standards.

Name	Quantitation transition			Qualifying transition #1			Qualifying transition #2		
	Precursor	Product	CE	Precursor	Product	CE	Precursor	Product	CE
^{13}C-PCB-111	338.00	268.00	25	266.00	196.10	30			
PCB-136	359.90	289.90	25	289.90	220.00	30			
PCB-110	325.90	256.00	25	254.00	184.00	30			
PCB-149	359.90	289.90	25	324.90	290.00	10			
^{13}C-PCB-118	338.00	268.00	25	266.00	196.10	30			
PCB-118	325.90	256.00	25	254.00	184.00	30			
^{13}C-PCB-153	372.00	302.00	25	302.00	230.10	30			
PCB-153	359.90	289.90	25	324.90	290.00	10	290.00	220.00	30
^{13}C-PCB-138	372.00	301.90	25	302.00	232.00	30			
PCB-138	359.90	289.90	25	359.90	324.90	10	289.90	220.00	30
PCB-187	393.80	323.80	25	358.90	323.90	10			
PCB-128	359.90	289.90	25	324.90	289.90	10			
^{13}C-PCB-180	405.90	335.90	25	336.00	266.00	30			
PCB-180	393.90	323.90	25	358.90	324.00	10			
PCB-200	429.80	359.90	25	357.90	287.90	30			
PCB-170	393.90	323.90	25	393.90	358.80	10	358.90	323.90	10
PCB-209	497.70	427.70	25	427.80	357.80	35			

Supplementary Table 4. Retention time (min) and recovery (%) of surrogate standards.

Name	Retention time (min)	^{13}C quantitation standard	Recovery [% ± SD]
			^{13}C-PCB 111
^{13}C-PCB-111	20.85	Recovery standard	100 ± 0
PCB-136	20.96	13C-PCB-138	
PCB-110	21.02	13C-PCB-118	
PCB-149	21.45	13C-PCB-153	
^{13}C-PCB-118	21.54	Quantitation standard	59.4 ± 10.5
PCB-118	21.55	13C-PCB-118	
^{13}C-PCB-153	21.93	Quantitation standard	57.9 ± 10.9
PCB-153	21.94	13C-PCB-153	
^{13}C-PCB-138	22.42	Quantitation standard	58.3 ± 10.7
PCB-138	22.42	13C-PCB-138	
PCB-187	22.69	13C-PCB-180	
PCB-128	22.89	13C-PCB-138	
^{13}C-PCB-180	23.63	Quantitation standard	57.9 ± 10.9
PCB-180	23.64	13C-PCB-180	
PCB-200	23.83	13C-PCB-180	
PCB-170	24.12	13C-PCB-180	
PCB-209	26.35	13C-PCB-180	

Supplementary Table 5. Method detection limit (MDL) in pg g^{-1} calculated as the mean blank concentration plus three times of standard deviation.

MDL (pg g^{-1}) (mean blank concentration + 3 x SD)				
**PCB #**	**Batch 1**	**Batch 2**	**Batch 3**	**Average**
PCB-136	0.077	0.045	0.059	0.060 ± 0.013
PCB-110	0.467	0.146	0.238	0.284 ± 0.135
PCB-149	0.205	0.230	0.238	0.224 ± 0.014
PCB-118	0.000	0.082	0.131	0.071 ± 0.054
PCB-153	1.634*	1.477	0.789	1.300 ± 0.367
PCB-138	0.387	0.203	0.263	0.284 ± 0.076
PCB-187	0.124	0.173	0.081	0.126 ± 0.038
PCB-128	0.112	0.073	0.059	0.081 ± 0.023
PCB-180	0.276	0.104	0.099	0.160 ± 0.082
PCB-200	0.000	0.000	0.000	0.000 ± 0.000
PCB-170	0.097	0.045	0.053	0.065 ± 0.023
PCB-209	0.361	0.241	0.419	0.340 ± 0.074

* indicates that an outlier was removed from the batch.

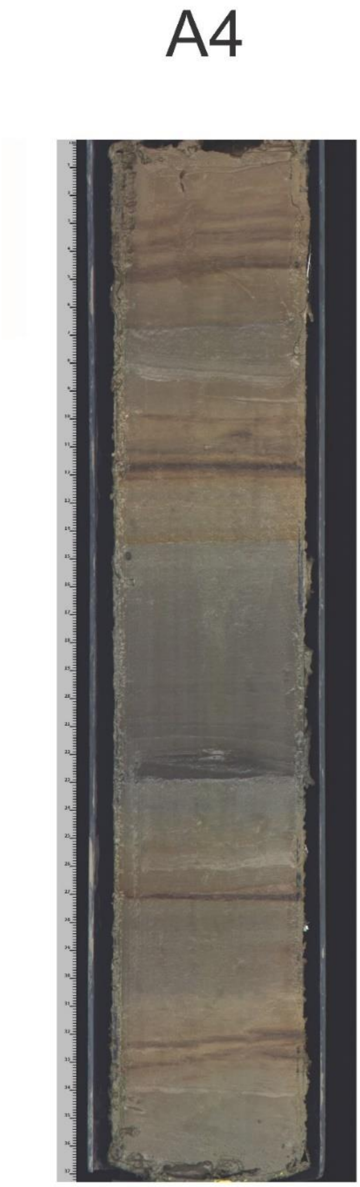

Supplementary Fig. 1. Photo of sediment core from Atacama 4. Distinct laminated structures in deeper layers suggest absence of bioturbation by infauna and occasional deposition events. Photo was taken by Matthias Zabel[1].

References

(1) Oguri, K.; Masqué, P.; Zabel, M.; Stewart, H. A.; MacKinnon, G.; Rowden, A. A.; Berg, P.; Wenzhöfer, F.; Glud, R. N. Sediment Accumulation and Carbon Burial in Four Hadal Trench Systems. *J. Geophys. Res. Biogeosciences* **2022**, 127, e2022JG006814. https://doi.org/10.1029/2022JG006814.
